# Reply to Verma et al. Comment on “Chao, H.-C. Zinc Deficiency and Therapeutic Value of Zinc Supplementation in Pediatric Gastrointestinal Diseases. *Nutrients* 2023, *15*, 4093”

**DOI:** 10.3390/nu16010136

**Published:** 2023-12-30

**Authors:** Hsun-Chin Chao

**Affiliations:** 1 Division of Pediatric Gastroenterology, Department of Pediatrics, Chang Gung Children’s Medical Center, Chang Gung Memorial Hospital, Taoyuan City 33305, Taiwan; hcchao1021@gmail.com; Tel.: +886-3-3281200; Fax: +886-3-3288957; 2 College of Medicine, Chang Gung University, Taoyuan City 33302, Taiwan

Your comment [1] on my article [2] is promising, especially regarding the awareness of zinc deficiency in adult celiac patients and the need of guidelines for appropriate therapeutic value to treat zinc deficiency in celiac patients.

The prevalence of zinc deficiency is remarkably high in celiac patients. Zinc deficiency was recognized to correlate with villous atrophy in celiac patients, and the risk of complications and mortality was increased in patients with persistent villous atrophy [3]. Regular assessment of nutritional status (including nutritional profiles) and dietary intake based on dietary records are crucial for celiac patients. Indeed, dietary zinc intake may vary depending on dietary pattern. As the beneficial effect of zinc as an adjuvant to a gluten-free diet in celiac disease remains controversial, it is worth establishing a human trial model to explore the impact of a zinc-rich diet or zinc supplementation on the recovery of intestinal villous atrophy and treatment outcome in celiac patients adopting a gluten-free diet.
